# Tools and Methods to Include Health in Climate Change Adaptation and Mitigation Strategies and Policies: A Scoping Review

**DOI:** 10.3390/ijerph18052547

**Published:** 2021-03-04

**Authors:** Ianis Delpla, Thierno Amadou Diallo, Michael Keeling, Olivier Bellefleur

**Affiliations:** 1 École Supérieure D’aménagement du Territoire et de Développement Régional (ESAD), Université Laval, Pavillon F-A. Savard, 2325, rue des Bibliothèques, local 1612, Québec, QC G1V 0A6, Canada; 2 National Collaborating Centre for Healthy Public Policy, Montréal, QC H2P 1E2, Canada; thiernoamadou.diallo@inspq.qc.ca (T.A.D.); michael.keeling@inspq.qc.ca (M.K.); olivier.bellefleur@inspq.qc.ca (O.B.)

**Keywords:** climate change policies, health, tools, adaptation, mitigation

## Abstract

Climate change represents a serious threat to the health and well-being of populations. Today, many countries, regions, and cities around the world are implementing policies and strategies to adapt to climate change and mitigate its effects. A scoping review was performed to identify tools and methods that help integrate health into climate change adaptation and mitigation policies and strategies. The literature search includes scientific and grey literature. The scientific literature was conducted using PubMed, Elsevier Embase, and Web of Science databases. A grey literature web search was performed to complement the results. A total of 35 studies (28 from the scientific literature and 7 from the grey literature) were finally included. A large majority of research articles (24/28) and almost all reports (6/7) from the grey literature were published after 2010. Results show that the tools that were found most frequently are the nested models (12/35), health impact assessment (6/35), vulnerability and adaptation assessment (3/35), conceptual frameworks (3/35), and mixed methods (3/35). This review shows an increasing interest in the topic of developing tools to better manage health issues in adaptation and mitigation strategies, with a recent increase in the number of publications. Additional analyses of tools’ effectiveness should be conducted in further studies.

## 1. Introduction

Climate change poses serious threats to human health and well-being, and it contributes to increasing health inequalities between and within countries. The health impacts of climate change can be direct (for example, heat waves, extreme weather, and events such as storms, forest fires, floods, or drought) or indirect through the effects of climate change on ecosystems (for example, agricultural losses, changes in disease distribution patterns, infrastructure disruptions) and on the economy and social structures (for example, migration and conflict) [1,2,3].

The health effects of climate change can be summarized as follows [4]: heat-related disorders, respiratory disorders, infectious diseases including vector- and water-borne diseases, disruptions to food production and mental health disorders. In 2014, World health Organization (WHO) established that more than 7 million deaths are attributable to air pollution each year [5]. WHO says climate change is likely to lengthen the transmission period of some major vector-borne diseases and change their geographic distribution. Concerning the effects on water-borne diseases, extreme weather events are associated with an increased risk of surface water contamination by protozoa, such as Giardia cysts and cryptosporidium oocysts [6]. Finally, the effects of climate change on mental health is an emerging research topic. These effects will not be felt in the same way by all, but will disproportionately affect vulnerable groups.

The 2015 Paris Agreement on Climate is seen as an important step towards the global strategy to reduce the risks of climate change [7]. This agreement called upon countries to take ambitious adaptation and mitigation actions and ensure their implementation [8]. Following this, the Intergovernmental Panel on Climate Change appealed for actions to limit the temperature increase to 1.5 °C to avoid, among others, significant risks to human health [9].

In this context, cities, and countries are developing policies or strategies to reduce greenhouse gas (GHG) emissions (mitigation) and to reduce vulnerability to the effects of climate change (adaptation). In these policies or strategies, it is necessary that health impacts be specifically taken into account in order to achieve both environmental protection and health promotion. However, health impacts are rarely considered by decision-makers involved in developing climate change adaptation and mitigation policies [10]. Actually, some existing tools such as health impact assessment (HIA) can be deployed to help take health issues into consideration when developing climate change-related policies [10].

However, to date and to our knowledge, there has not been a study that systematically reviews the tools and methods that were designed to promote or integrate health into climate change adaptation and mitigation policies or strategies. Selecting such tools and methods will help in the development of specific policies and strategies of adaptation and mitigation that contribute to environmental protection and health promotion.

The purposes of this paper are (1) to conduct a scoping review on climate change studies that focus on the development of tools and methods that help integrate health into climate change adaptation and mitigation policies and strategies, and (2) to assess and select the tools and methods that could be useful for stakeholders and contribute to protecting and promoting health.

## 2. Materials and Methods

### 2.1. Search Criteria

The literature search focused on the identification of all primary studies that aim to develop methods and tools that integrate health into climate change adaptation and mitigation policies and strategies published in English and French in scientific journals and grey literature between January 1990 and September 2019. The strategy used to conduct this review, in accordance with the PRISMA guidelines [11], consisted of grouping keywords that represented (i) the tools (frameworks, methods, etc.), (ii) the climatic events (namely, climate change and its effects: extreme events, etc.), and (iii) the health-related aspects (policies or diseases).

The following keywords and combinations were used for the literature search on Pubmed: (Tool*[TIAB] OR Method[TIAB] OR framework[TIAB] OR “Health Impact Assessment”[MeSH]) AND (“Climate change”[MeSH] OR “Climate change”[TIAB] OR “Climatic change”[TIAB] OR “Global warming”[TIAB] OR “Greenhouse effect”[MeSH:NoExp] OR “Extreme events”[TIAB]) AND (“Public health”[MeSH:NoExp] OR “Public policy”[MeSH:NoExp] OR “Health planning”[MeSH:NoExp] OR “Health policy”[MeSH:NoExp] OR “Air pollution” OR “Aeroallergens” OR “Heat waves” OR “Urban heat island effect” OR “Vector-borne diseases” OR “Water-borne diseases” OR “Water & food supply” OR “Mental health” OR “Environmental refugees”).

The literature search included scientific and grey literature. Keywords, titles, and abstracts were searched in PubMed, Elsevier Embase and Web of Science for scientific literature. For the grey literature search, Québec University databases, websites of the World Health Organization (WHO), the Intergovernmental Panel on Climate Change (IPCC), United Nations (United Nations Environment Programme (UNEP), United Nations Framework Convention on Climate Change (UNFCC)), World Meteorological Organization, ministries of health, and ministries of the environment of the United States, Canada (including state and provincial websites) and France were included. Considering the considerable number of documents published in the grey literature, the geographical area was restricted to North America to focus on regional studies and France to include some relevant studies published in French. There was no restriction on geographical location for the scientific literature search. Librarians of Laval University and of the documentation service of the Institut National de Santé Publique du Québec (INSPQ—Québec’s Public Health Institute) helped the two first authors to design and validate the literature search strategy.

### 2.2. Selection Criteria

In the first selection stage (exclusion criteria), the abstract of each article was read and screened. The papers meeting the following criteria were excluded from our review:

- Studies only presenting the climate change impacts on human health or the health system in qualitative or quantitative terms.
- Studies presenting only health impact assessment tools for extreme events (e.g., droughts, heatwaves, hurricanes).
- Studies presenting tools/methods that assess the health benefits of policies/strategies outside of the scope of climate change adaptation and mitigation.
- Commentaries, editorials, press releases, speeches, review articles, systematic reviews, or meta-analyses.
- Studies not published in English or French.

In a second selection stage (inclusion criteria), papers that remained from the previous stage were fully screened and then retained according to the following criteria:

- Studies that clearly present the link between the health issue and a meteorological factor that could be modified by climate change.
- Studies that aim to assess the health effects of a policy/strategy designed to adapt to or mitigate climate change consequences or reduce greenhouse gas emissions.
- Studies in which tools/methods are presented and described.

Finally, the references sections of the studies identified were screened, and relevant references that were not initially identified were added.

### 2.3. Data Extraction

Selected articles were reviewed and documented for the following information: first author, location, date of publication, type of climatic event, exposure measurement, health outcomes assessed, type and description of mitigation/adaptation strategy, tool name, origin, objective, and description; and strategy’s health effects measurements.

## 3. Results

### 3.1. Description of Studies Selected

#### 3.1.1. Scientific Literature

The literature search was conducted between 4–7 February 2019; 1718 articles were identified.

The first selection was based on titles, keywords, and abstracts. It should be noted that a majority of studies used models to assess the health impacts of different climate change scenarios without presenting any adaptation or mitigation strategies or tools and methods. These studies were not retained for in-depth review.

After this first selection, 108 articles were retained. Among these articles, 4 were unavailable, 2 were not in French or English, and 1 was a conference proceeding; these were consequently removed. Then, a final selection of 101 articles was retained for in-depth review.

#### 3.1.2. Grey Literature

The grey literature review was conducted between 15 February and 15 March 2019. The first selection was made based on titles, keywords, and abstracts. 43 reports were selected for in-depth review. All studies were in English. Table 1 synthesizes the scientific and grey literature search results.

**Figure 1:**
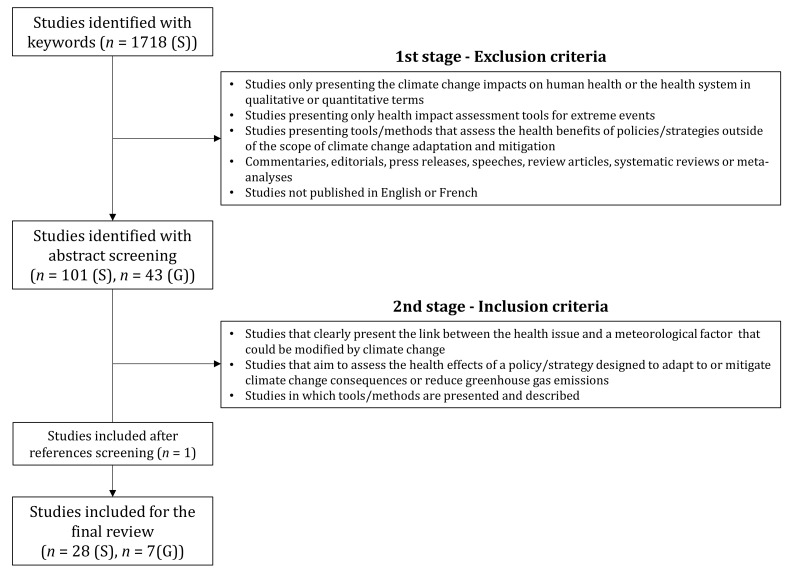
Data research results (S: Scientific literature; G: Grey literature).

The detailed results of the article and report selection are presented in Figure 1.

### 3.2. Description of Tools Selected

In the scientific literature, the studies identified were conducted mainly in Europe (11), followed by North America (7) and Asia (6), the remaining ones being conducted in Australia (1) and New Zealand (1). There was a majority of mitigation studies that focus on exposure to air pollution (22 of 28), such as particulate matter (PM2.5 and/or PM10). Some studies focus on exposure to air temperature (*n* = 3) or urban heat island (*n* = 2). The main health indicators used are mortality, disability-adjusted life years (DALY), and years of life lost (YLL). Publication dates were between 2008 and 2018, with a large majority of studies (24 of 28) published after 2010. Reports found through the grey literature research have different characteristics than the scientific literature. The WHO is the main organization providing tools aiming at including health in adaptation plans and policies (6 of 7 reports). The reports were published between 2003 and 2018, with almost all after 2010 (6 of 7).

The different tools that were identified through this literature review were grouped into four main categories: impact assessment tools, adaptation tools, nested models, and conceptual frameworks. We also added one additional category that regrouped mixed methodological approaches. The tools are presented in Table 2.

#### 3.2.1. Impact Assessment Tools

##### Health Impact Assessment (HIA)

HIA has been defined as “a combination of procedures, methods and tools by which a policy, programme, or project may be judged as to its potential effects on the health of a population, and the distribution of those effects within the population” [47]. It consists of five steps: (1) Screening: to decide whether or not a proposal needs an HIA; (2) Scoping: to plan how the HIA should be done; (3) Appraisal: to determine the extent to which the proposal will affect health, the nature of these effects and which population groups will be affected by these effects; (4) Recommendations and reporting: to formulate recommendations for minimizing the negative effects and maximizing the positive effects of a proposal and to present the results of the HIA in a report; (5) Evaluation and monitoring: to review the process and its influence and to monitor what happens once the proposal is put in place [47]. HIA can be used to analyze the potential health impacts of planned climate change policies. For example, this tool can be used to assess the impact on health of climate change mitigation policies at the local level [19,20], to evaluate the climate change resilience that is incorporated into specific building projects [24], or to study the health co-benefits of local climate change mitigation policies in the transport sector [29,34].

##### Comparative Risk Assessment (CRA)

The World Health Organization (WHO) defined Comparative Risk Assessment (CRA) as “the systematic evaluation of the changes in population health which result from modifying the population distribution of exposure to a risk factor or a group of risk factors” [48]. This method consists of four steps (adapted from [49]): (1) Identifying health risks associated with exposure; (2) Quantifying the dose-response relationship for a baseline; (3) Defining future exposure scenarios, and (4) Estimating the burden of disease that is attributable to a risk factor and the burden that is avoidable by plausible reductions in the risk factor. CRA has been used to inform climate change mitigation decisions [49]. For example, it has been used to evaluate the potential health co-benefits from the establishment of a mass rapid transit project in Kuala Lumpur [25]; to estimate the health effects of alternative urban land transport scenarios in the context of greenhouse gas emissions reduction in London and Delhi [38]; and to evaluate the health benefits resulting from GHG reduction measures in the electricity generation sector in the European Union (EU), China, and India [28].

##### Integrated Environmental Health Impact Assessment (IEHIA)

Integrated environmental health impact assessment (IEHIA) is an inclusive approach for assessing health-related issues associated with environmental changes in order to consider the complexities, interdependencies, and uncertainties of the real world [14]. As [14] indicated in his study, IEHIA derives from risk assessment, environmental impact assessment, HIA, and CRA. The IEHIA process involves four steps [14]: (1) Issue framing: specifying the policy question and developing a conceptual model of the issue to be addressed; (2) Design: defining a detailed protocol for assessment from the conceptual model elaborated in the previous step. This will include defining variables, causal relationships, policy scenarios, models, data, and tools. (3) Execution: modeling and analyzing different scenarios in terms of exposure and health effects and comparing results of the assessment; (4) Appraisal: this step involves synthesizing and interpreting the results; evaluating the outcome actions for the scenarios; and prioritizing the different policy options based on acceptability or effectiveness.

##### Environmental Assessments

The aim of environmental assessments is to identify and evaluate the potential consequences on the environment of proposed initiatives. In this context, environmental impact assessment (EIA) is used to assess the environmental impacts of projects while strategic environmental assessment (SEA) is applied to policies, plans, or programs. EIA and SEA processes are quite similar to that of HIA. EIA can be used to determine adaptation and mitigation measures that are favorable to both the environment and health. This implies that EIA is conducted in accordance with the European Union Directive (2014/52/EU), which broadened the scope of assessment by explicitly including human health among the topics to be addressed in an EIA. Similarly, SEA can be applied to select climate change adaptation or mitigation options to favor environmental protection and health promotion. To achieve this, conducting SEA needs to comply with the Directive 2001/42/EC of the European Parliament and of the Council and the SEA protocol to the Espoo Convention, both requiring human health issues to be taken into account when conducting a SEA.

#### 3.2.2. Adaptation tools

##### Vulnerability and Adaptation (V&A) Assessment

The WHO Executive Board has developed the Vulnerability and adaptation (V&A) assessment. This is an approach to understand the current and future health risks related to climate change and thus develop strategies, policies, and measure to better address these risks [46]. It is a way to help involve the health sector in climate change adaptation efforts [50].

The objective of a V&A assessment is to assist decision-makers in understanding the potential health risks attributable to climate change, in managing these risks, and in prioritizing policies and programmes to improve population health in a changing climate. A V&A assessment involves the following five basic steps: (1) Define the frame and scope of the assessment; (2) Conduct the vulnerability and adaptation assessment; (3) Understand future impacts on health; (4) Prioritize and implement health protection regarding adaptation to climate change; (5) Establish an iterative process for managing and monitoring the health risks of climate change [44].

An example of an adaption tool is the Geospatial Emergency Management Support System (GEMSS), a browser-based tool. GEMSS is a platform developed by the Texas Water Development Board for integrating data linked to environmental, public health, and policy indicators related to climate change that can be visualized on the web. It makes it possible to combine the information in the form of several maps: climatic events (hot days, heavy rains), mortality, an indicator of vulnerability, climate-related policies. This tool has been used by the City of Austin to assess climate-health vulnerability [23], including adaptation and mitigation policies.

##### Health National Adaptation Process (HNAP)

Developed by the WHO, and based on the United Nations Framework Convention on Climate Change (UNFCCC) climate change agenda, the HNAP’s objective is to ensure that the iterative management process of health risks resulting from climate change is integrated into the overall national adaptation plan (NAP) process to achieve the goal of a healthy population in healthy communities. This process consists of the following four elements (A–D), which are subdivided into eleven steps: 

(**A**) **Lay the groundwork and address gaps in undertaking the HNAP process:**

Step 1. Aligning the health adaptation planning process with the national process for developing a NAP;
Step 2. Taking stock of available information;
Step 3. Identifying approaches to address capacity gaps and weaknesses in HNAP implementation.

(**B**) **HNAP preparatory elements:**

Step 4. Conducting a V&A assessment in the health sector, including short- and long-term needs in the context of development priorities;
Step 5. Examining the implications of climate change for development goals, legislation, strategies, policies, and plans related to health;
Step 6. Developing a national health adaptation strategy that identifies priority adaptation options.

(**C**) **Implementation strategies:**

Step 7. Elaborating an implementation strategy for operationalizing HNAPs and incorporating climate change adaptation in health-related planning processes at all levels, including strengthening the capacity for conducting future HNAPs;
Step 8. Promoting coordination and synergies with the NAP process, especially with sectors affecting health, and with multilateral environmental agreements.

(**D**) **Reporting, monitoring, and review:**

Step 9. Following-up and reviewing the HNAP to assess progress, effectiveness, and gaps;
Step 10. Updating the health component of the NAPs in an iterative manner;
Step 11. Communicating and reporting on the progress and effectiveness of the HNAP implementation [45].

##### Economic Assessment Tool—Health and Adaptation Costs

The WHO Regional Office for Europe has developed this economic analysis tool to facilitate the planning of adaptation measures to protect health from the adverse effects of climate change in the Member States of the European Region. The tool allows users to estimate health and adaptation costs through three dimensions: (1) the costs related to damage to health caused by climate change; (2) the costs for adaptation in various sectors to protect health from such damage and (3) the efficiency of adaptation measures including avoided health costs [42]. The process for assessing health damage and adaptation costs involves four steps. Step (i) Define the scope of the assessment, involves deciding on the type of analysis and specifying the following elements: the level of application of the tool, the types of disease to be incorporated, the population groups concerned and the period of the analysis. Step (ii) Methods, data, sources, and analysis, consists of estimating both health damage costs and adaptation costs. It involves understanding methods, identifying data and sources, collecting data and inserting them in Excel sheets, conducting a sensitivity analysis, and analyzing results. Step (iii) Compare damage and adaptation costs, involves conducting a crude cost-benefit assessment. Step (iv) Present results, considers the target audience and communication needs.

#### 3.2.3. Nested Models

Nested models have been defined and used to assess the effect on health of greenhouse gas (GHG) reduction policies in different sectors (transport, energy, industry, or building), as well as in urban planning (greening policies, in [17]). The energy sector is the most studied, and nested models have been applied regionally to study the energy sector in different jurisdictions such as in China [16,28], Europe [28,36], and the United States [15]. The other applications are linked with building emissions [12,35], with public transport in municipalities [30], or in the cement industry [39]. Finally, some studies assess the global effects of national or subnational carbon policies [21,33,37]. These type of tools have the same general structure and consist of a suite of several nested modeling modules: different mitigation scenarios, a model converting these scenarios into modifications in GHG emissions (NOx, CO2, PM, or ozone), a model estimating resulting atmospheric pollutant concentrations, and finally a health effects assessment model (i.e., BenMAP, [12]). These ensembles of models also include some tools already developed, such as the Health Economic Assessment Tool, developed by the WHO to estimate economic and health benefits of different policies, or HIA and comparative risk assessment [28]. Nested models are tools that allow for the prediction of the health impacts of different adaptation or mitigation strategies, although these strategies are mainly hypothetical and based on global policy objectives (such as the Paris Agreement, for example).

#### 3.2.4. Conceptual Frameworks

This review of the scientific and grey literature revealed three examples of conceptual frameworks of interest as tools for integrating health into climate change adaptation/mitigation policies.

The first one is the “Ecosystems enriched” Driver, Pressure, State, Exposure, Effect, Action (eDPSEEA). Chiabai et al. [18] used the eDPSEEA framework to link climate change, adaptation actions and health by focusing on the potential health effects of changes in green spaces. In the proposed eDPSEEA conceptual framework, the driver is climate change caused by GHG emissions and concentrations. The pressures are represented by temperature and precipitation patterns, heat, air pollution and extreme weather events. This contributes to a potential change in the state of the environment, producing alterations in the functioning of the ecosystem that will then affect the terrestrial distribution of natural areas, and the regulation of the ecosystem services they provide in the short and long term. The state is characterized by 6 types of ecosystemic services that can affect the use or perception of a site through exposure (exposure): urban heat islands effect, air pollution, water cycle regulation, social environment, recreation, tourism, and microbiome. Based on a set of contextual factors (socioeconomic status, health status, culture, attitudes, beliefs, and environmental factors), these changes may have a direct or indirect, positive or negative impact on health (effect). Any intervention affecting green spaces and population exposure are considered an action.

The second one is a framework based on risk modelling to evaluate and provide scientific assessments of potential climate change adaptation measures. This framework includes three components: (1) knowledge synthesis; (2) Data storage and access; and (3) Quantitative risk modeling. Smith et al. [32] applied the framework to assess and compare the impacts of different adaptation scenarios related to water and food safety.

The third one is Climate, health and equity vulnerability assessment (CHEVA). CHEVA is a framework for assessing climate and health vulnerability. It includes four components for which a list of indicators is provided: (1) current and future physical threats of climate change; (2) population vulnerabilities including social determinants of health; (3) “adaptive capacity” that reflects individual and community-based resources that could counteract the negative impacts of climate change; and (4) health impact projections. A list of indicators has been developed to support the use of CHEVA. These indicators cover climate threats, population vulnerability, adaptive capacity, and resiliency [40].

#### 3.2.5. Other Methodological Approaches

This review of the scientific and grey literature revealed three examples of methodological approaches of interest as tools for integrating health into climate change adaptation/mitigation policies.

##### Participatory approach

The participatory approach is a way to mobilize and involve the community in the fight against climate change. This approach has been used to assess the effects on the quality of life and well-being of residents and users of several pilot projects on the reduction of urban heat islands in the Montréal area. The assessment was based on four criteria: beauty, comfort, coolness and security [13].

##### Mixed Methods

Mixed methods refer to methods that combine different analytical approaches (survey data, literature review, models, and tools). Haluza et al. [22] applied a mixed-methods approach in the energy sector to estimate the health impacts of a shift from light fuel to residential wood-burning in one state in Austria. The methodological approach combines modeling to estimate emissions of air pollutants with reviewing epidemiological studies to assess health impacts (literature review).

In the transport sector, Lindsay et al. [27] used a combination of survey data and tools to study the impact of shifting urban transport from cars to bicycles on health, air pollution, and GHG emissions, in New Zealand. The following data sources and tools were used: the New Zealand Household Travel Survey, the Vehicle Emissions Prediction Model, the Health and Pollution in New Zealand study, the WHO Health Economic Assessment Tool (HEAT), and the Cycling injury/death data and ‘safety in numbers’ [27].

Finally, Smith et al. [31] presented a set of scoping methods to quickly assess co-benefits for interventions in the energy sector.

## 4. Discussion

The tools that were found most frequently in this scoping review are the nested models (12 of 35); these were applied to assess the health impacts of mitigation options on different sectors such as transport, energy, industry, or building, as well as in urban planning. Health Impact Assessments were also frequently identified (6/35) and were applied to specific building projects, alternative transportation options or in urban planning.

Among the identified tools, several have a similar structure. For example, the family of impact assessments such as EIA, SEA, and HIA comprises similar tools to aid decision-making processes, but the tools differ in the scope of impacts analyzed. Environmental assessments (EIA and SEA) focus on the impacts of proposed policies or projects on the physical environment, while HIA analyzes the consequences of these policies and projects on physical, social, and economic determinants of health. EIA, SEA, and HIA are prospective tools based on methodologies that attempt to integrate the concerns of different stakeholders’ groups into the assessment process [47]. This means that an interdisciplinary and intersectoral scientific approach could be needed to evaluate the public health benefits of climate change adaptation or mitigation policies. Indeed, HIA is a combination of methods and tools. One of the differences between HIA and CRA is the object of analysis, which is a policy, program or project in the case of an HIA and a substance or exposure in the case of a CRA. A CRA can be integrated into an HIA framework.

The tools used could be different depending on the strategy chosen to face climate change consequences (adaptation or mitigation). Some tools tend to be dedicated to adaptation contexts such as V&A assessment, Health National Adaptation Process, or health and adaptation costs. In contrast, nested models are also almost exclusively (11/12 studies) used to assess mitigation scenarios in prospective studies.

The scale of application also differs among the different tools identified. Nested models are adaptable to varying scales of study, and this flexibility means that they are applied to both city, national-level, or supranational contexts, depending on the policy. Some tools such as vulnerability assessment, also allow an application at different scales. Some methods, such as HIA, are applied mostly at a local scale (city- or project-level). However, it should be noted that these tools are not designed specifically to be applied at a particular scale and might also be applied at other scales. It is worth noting that studies identified in the scientific literature are mainly conducted in wealthier nations and regions, in Europe, North America, and Asia. Few case studies have been conducted in developing countries. Some studies were conducted in India, Malaysia, and Thailand, but no studies were reported in Africa, the Middle East or in South America. However, these regions may suffer greater mortality risks due to climate change [51,52].

The robustness of data is also of primary importance to provide robust tools. Practitioners need to rely on robust data from health networks and emissions monitoring networks in place. Quantification of health impacts is often a difficult exercise due to the availability of valid data and rigorous analytical methods for predicting these impacts [53,54].

Some limitations in the application of these tools are linked with the accessibility of data. The use of impact assessment tools, adaptation tools, and nested models all depend upon access to a lot of different data relating to impact quantification, potential health impacts or scenario assessment, as well as data on climatic or health conditions or socioeconomic profiles of the population.

This review shows that in recent years there has been increased interest in the topic of developing tools to better manage health issues in adaptation and mitigation strategies. This subject emerged as a scientific topic at the end of the 2000s, with an increase in the number of publications in recent years. Nested models have also been increasingly used since 2014; these complete the other groups of tools that emerged in the 2000s.

In a context of a health crisis such as the Covid-19 pandemic, which has led to the lockdown of billions of people, to dramatic decreases in transport emissions and industrial emissions in the main GHG emitting countries [55,56,57], the methods identified in this review could provide an interesting basis for assessing the health co-benefits of such rapid mitigation measures. Some of the methods identified, such as nested models could allow for the assessment of the short-term health effects of a decrease in transport emissions in a region (such as in Lombardy, Catalunya, or in the greater Paris region) or across a greater geographical area such as a country (e.g., China, [58]) or an entire continent. From another perspective, some actions taken during the COVID-19 pandemic could be favorable to the climate. In this context, some tools identified in this work, such as environmental impact assessment and HIA, can help optimize the health benefits of these actions before their implementation with a view to combating climate change.

This literature review, as is often the case in bibliographic research, has some limitations. Despite a rigorous methodological approach, it is possible that some relevant articles were not identified. There are three reasons for this. The first is that the keywords used for the research may not have been included in the titles or abstracts of relevant documents. The second is the exclusion of publications in languages other than French and English. Finally, the third is that this literature review focused only on the documentation available online and thus excluded all other documents that were not published online. T One additional limitation is that the choice of keywords used in the literature search could have disproportionately favored some tools in the results, as for example, the term “health impact assessment” that was included in the keywords search. However, this keyword was selected in keeping with this review’s primary aim: to identify the tools that assess the health impacts of climate change. Removing this term from the literature review would have also led to missing some articles directly related to the objectives of this review. One limitation is that no analysis of the effectiveness of the tools identified was made, as that was judged to be beyond the scope of this review. Despite these limitations, we believe that this work helps to highlight several existing tools and methods that enable the integration of health concerns into the development of climate change adaptation and mitigation policies and strategies.

As suggested, an analysis of the effectiveness of these tools should be conducted in further studies. Moreover, the transferability of the tools identified in different jurisdictions (federal, provincial, or municipal) should be made in order to better identify the facilitating factors and the barriers for their application (such as, for example, political commitment, working in silos, knowledge, and skills). Uncertainties in climate change projections are linked with climate feedback, regional changes, or the strength of equilibrium climate sensitivity [59]. A better estimation and reduction of these uncertainties (and those linked with the quantification of health impacts) is also needed to refine and better approximate the quantification of health impacts.

## 5. Conclusions

Different countries, regions and cities around the world are implementing policies and strategies to adapt to climate change and mitigate its effects. This scoping review allowed for the identification of a total of 35 studies (28 from the scientific literature and 7 from the grey literature) that present tools and methods that were designed to promote or integrate health into climate change adaptation and mitigation policies or strategies. An increasing interest in this topic in the scientific literature was also noted since the beginning of the 2010s. The methods and tools that were most frequently found in this review are the nested models (12 of 35) and HIA (6 of 35) that were used to assess the health impacts of mitigation and adaptation options on different sectors such as transport, energy, industry or building, as well as in urban planning. Further studies should include additional analyses of the effectiveness of the tools and methods identified, as well as a quantification of the uncertainties linked with climate change projections and health impact estimates.

## Figures and Tables

**Table 1:** Scientific and grey literature review results, number of articles by language and database.

Type of Literature-	Source	Number of Documents	Total
Scientific literature	Web of science	575	1718 *
	Pubmed	525	
	Embase	982	
Grey literature	WHO	19	43
	IPCC	3	
	WMO	5	
	Ministry of Environment	British Columbia (1); United States (3); California (1)	
	Ministry of Health	Ontario (2); British Columbia (1); Canada (1); California (1); France (1)	
	Others	5	

* After duplicate suppression.

**Table 2:** (**a**) Synthesis of tools from the selected articles: scientific literature. (**b**) Synthesis of tools from the selected articles: grey literature.

**(a)**												
**Author**	**Year**	**Climatic Event**	**Exposure**	**Health Issue**	**Strategy (Adaptation/Mitigation)**	**Tool Type**	**Name**	**Provider**	**Tool’s Objective**	**Scale of Application**	**Area**	**Measure of the Health Effect**
Abel et al. [12]	2018	Rising temperatures	Air pollution (PM2.5 and ozone)	Incidence of premature mortality and morbidity	Mitigation	Nested models	EPA’s BenMAP Community Edition version 1.3	US EPA	BenMAP calculates adverse health outcomes of air quality changes linked with adaptation in building energy use	Region	East and Midwest USA	Yes
Beaudoin et Gosselin [13]	2016	Rising temperatures	Urban heat islands	Well-being	Adaptation	Other methodological approach	UNSP	National Institute of Public Health of Québec	Assess the effects of Urban Heat Island on well-being and quality of life of residents and users. 4 criteria assessed: beauty, comfort, coolness, and security	City	Montréal (Canada)	No
Briggs [14]	2008	UNSP	UNSP	UNSP	UNSP	Impact assessment tool	Integrated environmental health impact assessment	Imperial College London	Assess health-related issues deriving from the environment, and health-related impacts of policies and other interventions that affect the environment, taking into account complexities, interdependencies and uncertainties of the real world	Different scales (local to global)	UNSP	Yes
Buonocore et al. [15]	2016	UNSP	Air pollution (PM2.5, NOx and SO2)	Premature deaths	Mitigation	Nested models	UNSP	Harvard University	Assess reductions in NOx, PM2,5 and CO2 and health gains associated (Premature deaths avoided per year) with offshore wind electricity	States	New Jersey and Maryland (USA)	Yes
Cai et al. [16]	2018	UNSP	Air pollution (PM2.5)	Premature deaths	Mitigation	Nested models	UNSP	Joint Center for Global Change Studies	Assess reductions in NOx, PM2,5 and CO2 and associated health gains of carbon dioxide mitigation in the electric power generation sector	Country (subregions)	China	Yes
Chen et al. [17]	2014	Heatwaves	Air temperature	Heat-related mortality	Mitigation	Nested models	UNSP	CSIRO	Assess the impact of urban vegetation in the reduction of heat related mortality rate	City	Melbourne (Australia)	Yes
Chiabai et al. [18]	2018	Heatwaves, floods and heavy rainfalls	Urban heat islands, floods and air pollution (3 classes)	Multiples (non-specific)	UNSP	Conceptual framework	“Ecosystems enriched” Driver, Pressure, State, Exposure, Effect, Action (eDPSEEA)	BC3-Basque Centre for Climate Change, Spain	Linking CC impacts and adaptation actions on environment and assess how these actions could affect human health through various ways of exposure	UNSP	UNSP	UNSP
Diallo et al. [19]	2016	UNSP	Air pollution (PM10, NOx), noise	Disability adjusted life years (DALY) of sleep disorders and annoyance	Mitigation	Impact assessment tool	Health Impact Assessment	WHO	Assess the effects on health and well-being of greenhouse gases (GHG) reduction measures	City	Geneva (Switzerland)	Yes
Diallo et al. [20]	2017	UNSP	Air pollution (PM10, NOx), noise	DALY of sleep disorders and annoyance	Mitigation	Impact assessment tools	Health impact assessment (HIA), Other environmental assessment tools *	WHO (HIA), Conseil fédéral Suisse (SA)	Assess the impacts of different GHG reduction measures	City	Geneva (Switzerland)	Yes
Garcia Menendez et al. [21]	2015	UNSP	Air pollution (Ozone, PM2.5)	Mortality	Mitigation (GHG reduction scenarios)	Nested models	UNSP	Massachusetts Institute of Technology	Allow an integrated analysis of the effects of CC mitigation measures on air pollution and health co-benefits	Country	United States	Yes
Haluza et al. [22]	2012	UNSP	Air pollution (PM10, NOx)	Cardiovascular and respiratory mortality	Mitigation (scenarios)	Other methodological approach	UNSP	Institute of Environmental Health, Center for Public Health, Medical University of Vienna		Region	Upper Austria	Yes
Houghton et al. [23]	2012	Heatwaves, floods	Air temperature	Mortality (Cardiovascular, diabetes and hypertension)	Adaptation and Mitigation	Adaptation tools	Geospatial Emergency Management Support System (GEMSS)	Texas Water Development Board		City	Austin (USA)	No
Houghton [24]	2011	Tornadoes, hurricanes, heat/drought, and lightning	Air temperature, wind	Mortality, injuries	Adaptation and Mitigation	Impact Assessment Tool	Health Impact Assessment	WHO	Assess climate change resilience in specific building projects	City	Houston (USA)	No
Kwan et al. [25]	2016	UNSP	Air pollution (PM2.5), physical activity, and road crashes	Mortality	Mitigation	Impact Assessment Tool	Comparative Risk Assessment	WHO	Assess the co-benefits of a mass rapid transit project in terms of mortality reduction	City	Kuala Lumpur (Malaisia)	Yes
Li and Crawford-Brown [26]	2011	UNSP	Air pollution (PM2.5 and PM10)	Cardiovascular and respiratory (asthma, bronchitis) mortality	Mitigation	Adaptation tools	UNSP	US EPA	Support decision-making using cost-benefit comparisons and health co-benefit assessments of air pollution reduction	City	Bangkok (Thailand)	Yes
Lindsay et al. [27]	2011	UNSP	Air pollution (PM_10_, NO2, CO), physical activity, road crash	Cardiovascular and respiratory (bronchitis) mortality	Mitigation	Other methodological approach	Combination of tools and survey data	University of Auckland	Estimate the effects on health, costs, air pollution, GHG emissions if short trips were undertaken by bicycle rather than car	Country	New Zealand	Yes
Markandya et al. [28]	2009	UNSP	Air pollution (CO2, PM2.5)	Mortality (cardiorespiratory disease and lung cancer), acute respiratory infections	Mitigation	Nested models	Three models (POLES, GAINS, and WHO Comparative Risk Assessment)	WHO	Assess modifications of particulate air pollution and health effects resulting from GHG reduction measures in the electricity generation sector	Countries	European Union, China and India	Yes
Perez et al. [29]	2015	UNSP	Air pollution (PM2.5, elemental carbon), physical activity, noise	Mortality (noise, air pollution); DALY	Adaptation	Impact assessment tools	Health Impact Assessment	WHO	Assess the health impacts of local CC mitigation policies in the transport sector	City	Bâle (Switzerland)	Yes
Sarigiannis et al. [30]	2017	UNSP	Air pollution (PM2.5, PM10, NO2, and benzene)	Mortality, DALY	Mitigation (GHG reduction)	Nested models	UNSP	Aristotle University of Thessaloniki	Assess health co-benefits associated with GHG reduction policies in transportation	City	Thessaloniki (Greece)	Yes
Smith and Haigler [31]	2008	Rising temperatures	Air pollution (methane, CO2)	DALY, years of life lost (YLL)	Mitigation	Other methodological approaches	UNSP	WHO	Assess health co-benefits associated with GHG reduction policies in the energy sector	Country	China	Yes
Smith et al. [32]	2015	Rising temperatures	Water and food	DALY	Adaptation	Conceptual framework	UNSP	Public Health Agency of Canada	Provide a scientific assessment of CC adaptation measures to support risk management of climatic events	Region	Hypothetical case	Yes
Thompson et al. [33]	2016	UNSP	Air pollution (ozone and PM2.5)	Mortality risk, morbidity (hospital admissions, emergency room visits, lost school days, acute respiratory symptoms, acute myocardial infarction (nonfatal heart attacks) and acute bronchitis)	Mitigation	Nested models	UNSP	US EPA (BenMAP)	Assess health and monetary impacts of a carbon policy at the subnational scale	Region	Northeast USA (17 States)	Yes
Tobollik et al. [34]	2016	UNSP	Air pollution (PM2.5, elemental carbon) and noise	YLL, years lived with disability (YLD)	Mitigation	Impact assessment tool	Health Impact Assessment	WHO	Assess the health co-benefits of local CC mitigation policies in the transport sector	City	Rotterdam (Netherlands)	Yes
Tuomisto et al. [35]	2015	UNSP	Air pollution (PM2.5)	Mortality, DALY	Mitigation	Nested models	Opasnet	URGENCHE (EU FP7 project)	Estimate health impacts of emissions due to heat and power consumption of buildings and give guidance on different climate mitigation options	Cities	Bâle (Switzerland), Kuopio (Finland)	Yes
Williams et al. [36]	2018	UNSP	Air pollution (PM2.5, NO2 and ozone)	YLL	Mitigation	Nested models	UNSP	King’s College	Assess health co-benefits of different CC mitigation actions in the energy sector	Country	Great Britain	Yes
Wolkinger et al. [37]	2018	UNSP	Air pollution (PM2.5, PM10 and NO2)	Mortality, hospital admissions, and years lived with disability for cardiovascular and respiratory diseases. Physical activity.	Mitigation	Nested models	UNSP	Center for Climate and Global Change, (Austria)	Allow a detailed health and macroeconomic assessment of CC adaptation policies	Cities	Graz, Vienna and Linz (Austria)	Yes
Woodcock et al. [38]	2009	UNSP	Air pollution (PM2.5, PM10)	YLL, YLD, DALY, and mortality	Mitigation	Impact assessment tool	Comparative Risk Assessment	WHO	Compare the health effects of different mitigation scenarios with a reference situation	Cities	New Delhi (India); London, (United Kingdom)	Yes
Zhang et al. [39]	2016	UNSP	Air pollution (PM2.5)	Mortality and morbidity	Mitigation	Nested models	UNSP	Copernicus Institute of Sustainable Development (Utrecht University)	Assess the potential for energy savings and emission mitigation of air pollution from China’s cement industry, and quantify the health co-benefits linked with air pollution reduction in this sector	Regions	China (all provinces)	Yes
**(b)**												
**Author**	**Year**	**Climatic Event**	**Exposure**	**Health Issue**	**Strategy (Adaptation, Mitigation)**	**Tool Type**	**Name**	**Provider**	**Tool’s Objective**	**Scale**	**Area**	**Measure of the Health Effect**
Rudolph et al. [40]	2018	UNSP	UNSP	UNSP	Mitigation	Conceptual framework	Climate, health, and equity vulnerability assessment	Public Health Institute Center for Climate Change and Health	Assess health and climate vulnerabilities	UNSP	UNSP	UNSP
UNFCCC [41]	2011	UNSP	UNSP	UNSP	UNSP	Impact assessment tool	Health Impact Assessment	WHO, Curtin University WHO Collaborating Centre	Assess potential CC impacts and develop adaptation responses to support governmental decision making	UNSP	UNSP	Yes
WHO (Europe regional office) [42]	2013	UNSP	UNSP	UNSP	Adaptation	Adaptation tools	Health and adaptation costs	WHO	Support health adaptation planning in European states by estimating health and adaptation costs and efficiency of adaptation measures	Country	Europe	Yes
WHO [43]	2003	UNSP	UNSP	Mortality morbidity	Adaptation and Mitigation	Impact assessment tool	Quantitative health impact assessment	WHO	Quantify the burden of disease from specific risk factors and estimate the benefit of realistic interventions that remove or reduce risk factors	UNSP	UNSP	Yes
WHO [44]	2013	UNSP	UNSP	UNSP	Adaptation	Adaptation tools	Vulnerability and adaptation assessment	WHO	Provide guidelines to improve the elaboration of vulnerability and adaptation assessment and plan the adaptation of the health sector (similar to Health National adaptation process)	Country	UNSP	Yes
WHO [45]	2014	UNSP	UNSP	UNSP	Adaptation	Adaptation tools	Health National adaptation process	WHO	Ensure that the process of iteratively managing the health risks of climate change is integrated into the overall National Adaptation Plan process to achieve the goals of healthy people in healthy communities	Country	Directed to developing countries and least-developed countries	Yes (indicators)
Ontario government [46]	2016	UNSP	UNSP	UNSP	Adaptation	Adaptation tools	Vulnerability and adaptation assessment	WHO	Support a resilient and adaptive public health system to anticipate, take into account, and attenuate the emerging risks and impacts of CC (similar to National health adaptation process)	Province	Ontario, Canada	Yes

WHO: World Health Organization. UNSP: unspecified. * environmental impact assessment (EIA), strategic environmental assessment (SEA), sustainability assessment (SA). UNFCCC: United Nations Framework Convention on Climate Change.

## Data Availability

Data sharing not applicable.
